# Therapeutic Notes

**Published:** 1898-08

**Authors:** W. J. Martin

**Affiliations:** Kankakee, Ill.


### THERAPEUTIC NOTES.

Conducted by Dr. W. J. Martin,
KANKAKEE, ILL.

Bovine Tuberculosis. The application of the tuberculin-test to 308 cows within the city limits of New York has revealed the existence of tuberculosis in 52 head, a ratio of nearly 17 per cent. The milk-supply of 325 families was derived from two herds, comprising a total of 46 cows, in which examination demonstrated the presence of bacillus tuberculosis in 19. A postmortem examination in each of these cases showed that not in a single instance did the test prove false.

Antitoxin Treatment of Diphtheria. In 10,000 cases of diphtheria treated in 86 hospitals in America, Australia, and Japan with antitoxin the mean mortality was 18 per cent., against a previous mortality of 44.3 per cent. without antitoxin. In private practice there have been collected accounts of 3760 cases of diphtheria treated with antitoxin, with a death-rate as low as 7.8 per cent.

Ancient Scales. In recent excavations in the ruins of ancient Egypt there have been found several scales that are almost identical with the small hand prescription scale used in pharmacy at the present day.

Percentage of Quinine in the Different Salts of the Alkaloids. The following table, prepared by Tauret, is worthy of preservation :

| | Per Cent. |
|---|---|
| Acetate | 87.34 |
| Hydrate quinine, precipitated and dried | 85.70 |
| Basic chlorhydrate | 81.60 |
| Lactate | 78.26 |
| Basic bromohydrate | 76.60 |
| Valerianate | 76.05 |
| Basic sulphate (ordinary sulphate) | 74.30 |
| Sulpho-vinate | 72.00 |
| Neutral bromohydrate | 60.00 |
| Neutral sulphate, or acid sulphate | 57.24 |
| Tannate | 20.60 |

—*Myer Bros.' Druggist.*

Fossil Bacteria. M. Renault, in a paper read to the Academy of Science of Paris, says that he has found micrococci and bacilli fossil in the coal measures of St. Etienne and Commentary. The bacteria were most often found preserved in nodules of flint and in deposits of carbonate of lime. He also found them among the remains of partially carbonized plants of the coal period. In his communiction to the academy M. Renault figures and describes the remains of these minute microörganisms which he claims to have discovered.

The Tallow Tree. In the interior of Africa there grows a "tallow tree," the fruit of which contains seeds that are very rich in an extractive fatty matter which, when expressed, is made into candles that are extensively used by the natives for light-giving purposes and for barter with surrounding tribes.

Orthoform. A local anæsthetic for wounds, burns, and cancerous ulcers. This agent is a new synthethic derivative, is a methyl-ester of paraamidometa-oxybenzoic acid. It is a white crystalline powder, odorless, tasteless, and only slightly soluble in water. To this partial insolubility is due the advantage of this drug over other anæsthetics, as but a small amount of the drug is absorbed by the cutaneous tissues at a time; its anæsthetic action may thus be prolonged for several hours or even days.

A hydrochloric of orthoform is obtained by the combination of hydrochloric acid with orthoform. This combination occurs in the form of crystals which are very soluble in water, but, being acid, is not suitable for hypodermatic use, although its anæsthetic properties are not less active locally than orthoform.

Orthoform is said to be absolutely nontoxic. As much as sixty grains were given to rabbits per os for several days in succession. Dogs took as high as ninety grains at a dose, while forty-five grains were given hypodermatically, all without toxic effect. Along with its anæsthetic action, orthoform is an active antiseptic, preventing putrefaction and fermentation, and arrests their progress when present. It is best used in the form of a powder sprinkled evenly over the surface of the wounds.

An ointment of orthoform, for the dressing of painful wounds and burns, of the following formula is highly recommended: ℞. Orthoform, 96 grains; Adeps et lanolin, of each ℥iv. M.

Owing to its high price ($2.00 per ounce), orthoform is not likely to come into general use in veterinary practice.

REMOVAL OF BLOODSTAINS. Bloodstains may be removed from the clothing by washing in a weak solution of tartaric acid and afterward rinsing in clear water.

ANTISEPTIC POWDER. Iodoform, finely powdered, 930 gm.; Benzoin gum, finely powdered, 930 gm.; quinine, finely powdered, 960 gm.; magnesium, finely powdered, 930 gm.; oil eucalyptus, 120 gm.—*A. J. Pharmacy.*

POTENCY OF CRUDE DRUGS. Crude drugs vary greatly in their actions, some being potently active in their physiological action, others again being inert and useless. Great care should therefore be exercised in the purchase of crude drugs, powdered or otherwise. Cantharides, for instance, are often deprived of their physiologically active constituent, cantharidin, by unscrupulous manipulators by steeping the powdered drug in successive baths of chloroform. Commercial oil of eucalyptus is often so badly adulterated as to consist almost wholly of oil of turpentine of a very inferior quality. The writer once nearly lost an arm from septicæmia while depending, all unaware, upon an adulterated oil of eucalyptus as an antiseptic.

SUNFLOWER-SEEDS AS A POULTRY-FOOD. Sunflower-seeds rank high as a food for fattening poultry for market, giving the plumage a remarkable bright and healthy appearance, hence this food is of especial value for feeding to poultry used for exhibition purposes. Owing to a certain amount of oil contained in the seeds, it is an excellent food to feed to fowls during the moulting period. The seeds also increase the egg-producing capacity of ordinary hens.

LIQUID OLEATE OF AMMONIA LINIMENT. ℞.—Ammonia water, 100 gm.; lard oil, 90 gm.; cottonseed-oil, 110 gm. Mix by agitation in a bottle. This liniment is of the same ammoniac strength as liniment of ammonia U. S. P., and contains 57 per cent. of ammonium oleate which is perfectly saponified.—*Bull. Pharmacy.*

EVERY veterinarian's office should contain a well-stocked pharmacy, together with all the necessary utensils for the proper conduct of the same. These utensils should be kept scrupulously clean and in perfect order, so that each shall be readily available when wanted.

FORMALDEHYDE. The results of all investigations have led to the following conclusions :

1. Formalin in concentrated solution 1 : 10,000 makes the growth of tuberculosis, anthrax, cholera, typhus, pus, and diphtheria germs impossible.

2. In gaseous form a weak dilution is sufficient to check the growth of fungi.

3. A 1 per cent. solution will kill pathogenic organisms in an hour.

4. With a 3 per cent. solution and the final addition of alcohol it is possible to make the hands germ-free. Whether the skin of the hands is attacked by this method remains to be proved.

5. The spraying with formalin solution and the subsequent inclosure of articles in a closed space will easily sterilize them.—E. A. DE SCHWEINITZ, Ph.D., M.D.

As a local anæsthetic for hypodermatic use in the removal of tumors, a combination of cocaine, morphine hydrochlorate, and sodium chlorate gives good results. The manufacturing pharmacists now supply the above in tablet form.

Cottonseed Oil in two-ounce doses given three times a day mixed in oats is an excellent remedy to relieve and cure the irritable cough and sore-throat of horses suffering from influenza.

Sulpho-carbolate of zinc in drachm-doses given three times a day in warm water is a reliable remedy in acute or chronic dysentery of horses, more especially if the discharges are fetid in odor.

A solution consisting of 18 grains of bichloride of mercury in 10 ounces of lime-water is an efficient application in poisoning by Rhus toxicodendron.

It is said that the odor of iodoform may be removed from the hands and clothing by washing the hands and sponging the clothing with orange-flower water.

Formalin has been found to be an efficacious antidote to the stings of insects, the bites of serpents and small animals, such as dogs, cats, etc. The method of application is with a small brush, and after the evaporation of the formalin it may be reapplied. The effects are most soothing, pain is relieved, and inflammation rarely occurs after its use.

Apomorphine is a prompt and reliable antidote in strychnine-poisoning in dogs. It can be administered either hypodermatically or by the mouth. The tablets put up for use in human practice answer well for this purpose.

Where slow cutaneous absorption of a drug is desired in the form of an ointment, petrolatum should form the base. Where rapid absorption is desired, lanolin should be used as a base.

Sodium salicylate and antipyrine in combination are incompatible.

